# A Case of Postpartum Pulmonary Edema With Preserved Ejection Fraction and Diastolic Capacity

**DOI:** 10.7759/cureus.31179

**Published:** 2022-11-07

**Authors:** Shohei Tanabe, Sachiyo Sugino, Kotaro Ichida, Kiyoshi Niiya, Syuji Morishima

**Affiliations:** 1 Obstetrics and Gynecology, Kobe City Medical Center West Hospital, Kobe, JPN

**Keywords:** diagnosis, anemia, ultrasonography, differential diagnosis, postpartum pulmonary edema, heart failure

## Abstract

A 21-year-old female patient delivered vaginally at 40 weeks of gestation after an uneventful pregnancy. She bled profusely during delivery and underwent a blood transfusion and was discharged home on postpartum day 6. On postpartum day 8, she developed respiratory distress and visited our emergency room. She was admitted after a computed tomography scan showed evidence of pulmonary edema; pneumonia was suspected. After admission, her oxygenation worsened, and she was transferred to a higher institution. A PCR test performed at the higher institution was negative for coronavirus disease 2019, and echocardiography showed that the patient's ejection fraction was maintained. Oxygenation improved with oxygen administration alone, and the patient was transferred to our hospital on the same day. Echocardiography performed at our hospital showed no abnormalities in diastolic function, but the left ventricle was enlarged and mild mitral regurgitation was observed. Oxygenation gradually improved with diuretics and oxygen administration, and the patient was discharged home on the fifth day of hospitalization. An echocardiogram performed three months postpartum was normal.

## Introduction

Perinatal cardiomyopathy is a rare condition in which a healthy woman develops left ventricular dysfunction and heart failure during the perinatal period [1]. Diagnostic criteria for perinatal cardiomyopathy include a decreased ejection fraction (EF) of less than 45% on echocardiography [2]. Recently, however, cases of peripartum cardiomyopathy have been reported in which EF is preserved but left ventricular diastolic dysfunction develops, resulting in heart failure [3]. In this report, we describe a case of postpartum heart failure with normal EF and no left ventricular diastolic dysfunction but with transient left ventricular enlargement and mitral regurgitation (MR).

## Case presentation

A 21-year-old female patient with G1P0 conceived spontaneously and underwent a prenatal checkup at our hospital. She had no previous medical history and no history of cardiac disease. The pregnancy was uneventful. A blood test at 36 weeks five days gestation revealed a hemoglobin (Hb) of 9.9 g/dl. She delivered spontaneously by vaginal delivery at 40 weeks and 0 days. During delivery, a laceration occurred up to the posterior vaginal vault, resulting in 1577 g of blood loss. Blood was drawn, and the Hb level dropped to 5.3 g/dl. Red blood cell (RBC) 4U was transfused, and the Hb level improved to 7.1 g/dl. She was discharged home on a postpartum day 6. On the morning of the 8th postpartum day, she developed respiratory distress. As her symptoms did not improve, she visited the emergency department of our hospital. At the time of the visit, her vital signs were Blood Pressure (BP) 134/85 mmHg, Heart Rate (HR) 105 bpm, Oxygen saturation of peripheral artery (SpO2) 95% (room air), and Body Temperature (BT) 37.1℃. On auscultation, fine craquelure was heard bilaterally on both sides. Hb was 7.3 g/dl. The results of the blood test after a visit to our clinic are shown in Table 1.

**Table 1:** Results of blood tests performed at our hospital WBC: white blood cell; Hb: hemoglobin; Plt: platelet; Alb: albumin; AST: aspartate aminotransferase; ALT: alanine aminotransferase; LD: lactate dehydrogenase; BUN: blood urea nitrogen; Cre: creatinine; Na: sodium; K: potassium; Cl: Chlorine; CRP: C-reactive protein.

	Day 0	Day 1	Day 2	Day 4
WBC (/μl)	122	119.9	116.7	90.9
Hb (g/dl)	7.3	7.9	9	8.6
Plt (/μl)	35.6	38.1	41.3	51.7
Alb (g/dl)	2.77	2.63	2.92	2.92
AST (U/L)	21	20	18	15
ALT (U/L)	21	31	32	22
LD (U/L)	345	342	427	285
BUN (mg/dl)	8	7	9	11
Cre (mg/dl)	0.76	0.59	0.52	0.47
Na (mEq/l)	144	141	143	142
K (mEq/l)	3.6	3.8	4.2	4.2
Cl (mEq/l)	114	105	108	111
CRP (ng/ml)	1.07	6.75	5.25	1.47

**Figure 1:**
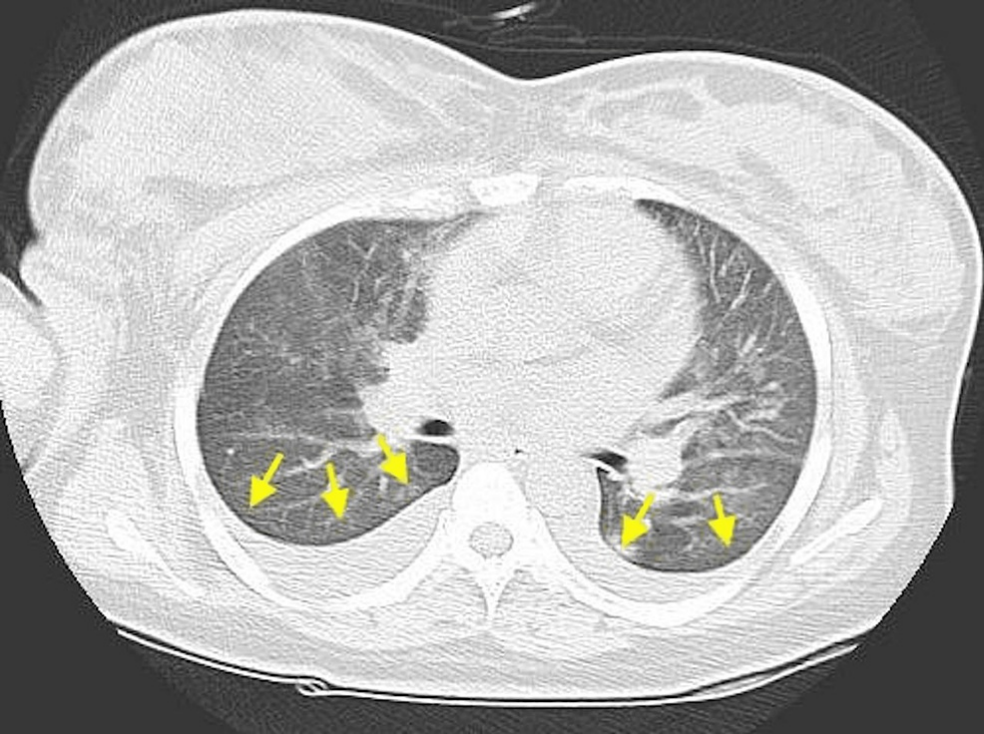
CT image of the chest Bilateral pleural effusions (yellow arrows)

**Figure 2:**
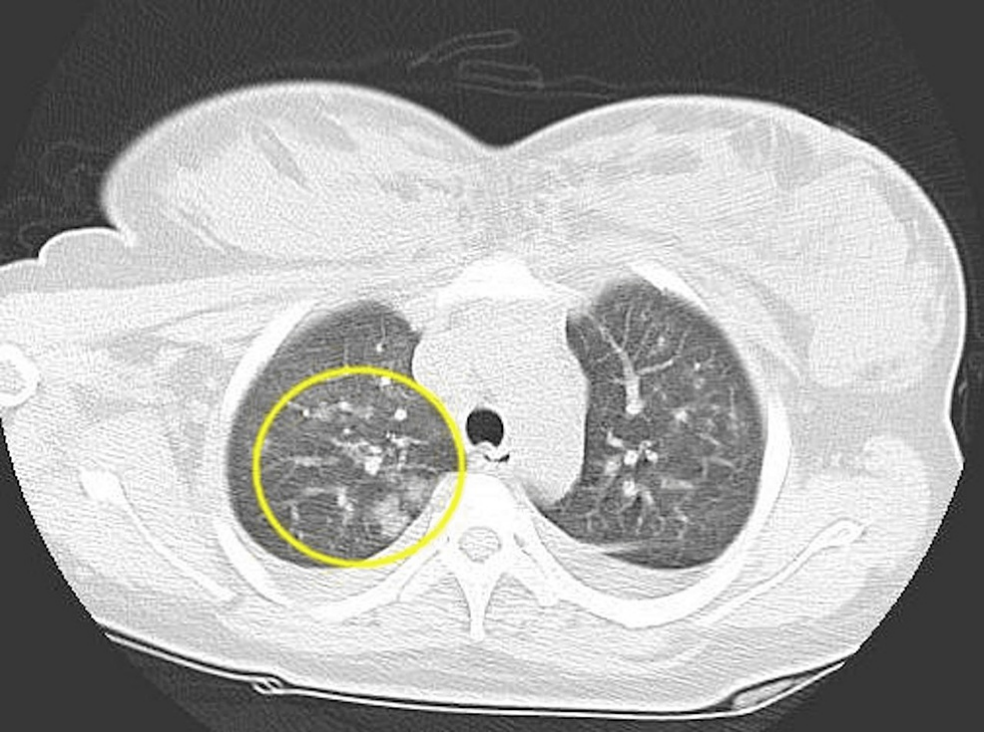
CT image of the chest Frosted shadows in the right upper lung field (yellow circle)

Chest computed tomography (CT) showed increased vascular shadows and pleural effusion in the bilateral lung fields (Figure 1) and frosted shadows in the upper lung fields (Figure 2). Since pneumonia was suspected, treatment with ceftriaxone was started. As the patient had not been vaccinated against coronavirus disease 2019 (COVID-19), the possibility of pneumonia caused by COVID-19 was also considered, and the patient was admitted to the isolation room after submitting a polymerase chain reaction (PCR) test. Since more than 6 hours had passed since the transfusion, TACO (Transfusion-associated circulatory overload) was considered negative. Four hours after admission, oxygenation worsened to the point that the patient required 5L of oxygen via a mask. The patient was transferred to a higher facility where intensive care was available. The PCR performed at the new hospital was negative. A second PCR test administered at the hospital also showed negative results, and the diagnosis of pneumonia caused by COVID-19 was ruled out.

The patient was transported back to our hospital after oxygenation improved only with oxygen administration, and the oxygen volume was reduced to 2L via a nasal cannula. Echocardiography performed at the higher institution showed EF 55%~60%, preserved cardiac output, and negative troponin. Echocardiography performed at our hospital showed a slightly enlarged left ventricular dimension (LVD) of 53 mm (normal value: 39-55 mm) and mild MR. Early diastolic mitral annular velocity (E`) was 8.8 cm/s, the ratio of the early transmitral flow velocity and the early mitral annular velocity (E/E`) was 12.1, left atrium (LA) volume index was 28.2 ml/m^2^, and tricuspid regurgitation (TR) was trivial, but TR velocity peak was 2.8 m/s, and heart failure with preserved ejection fraction (HFpEF) was 55%~60%. The diagnostic criteria for HFpEF were not met. Echocardiography showed preserved EF and no impairment of diastolic function, but the presence of pulmonary edema, leg edema, and an elevated brain natriuretic peptide (BNP) level of 170.6 pg/ml led to the diagnosis of heart failure. Continuous administration of furosemide 20 mg/day and 1 L of oxygen was started as a treatment for heart failure.

**Figure 3:**
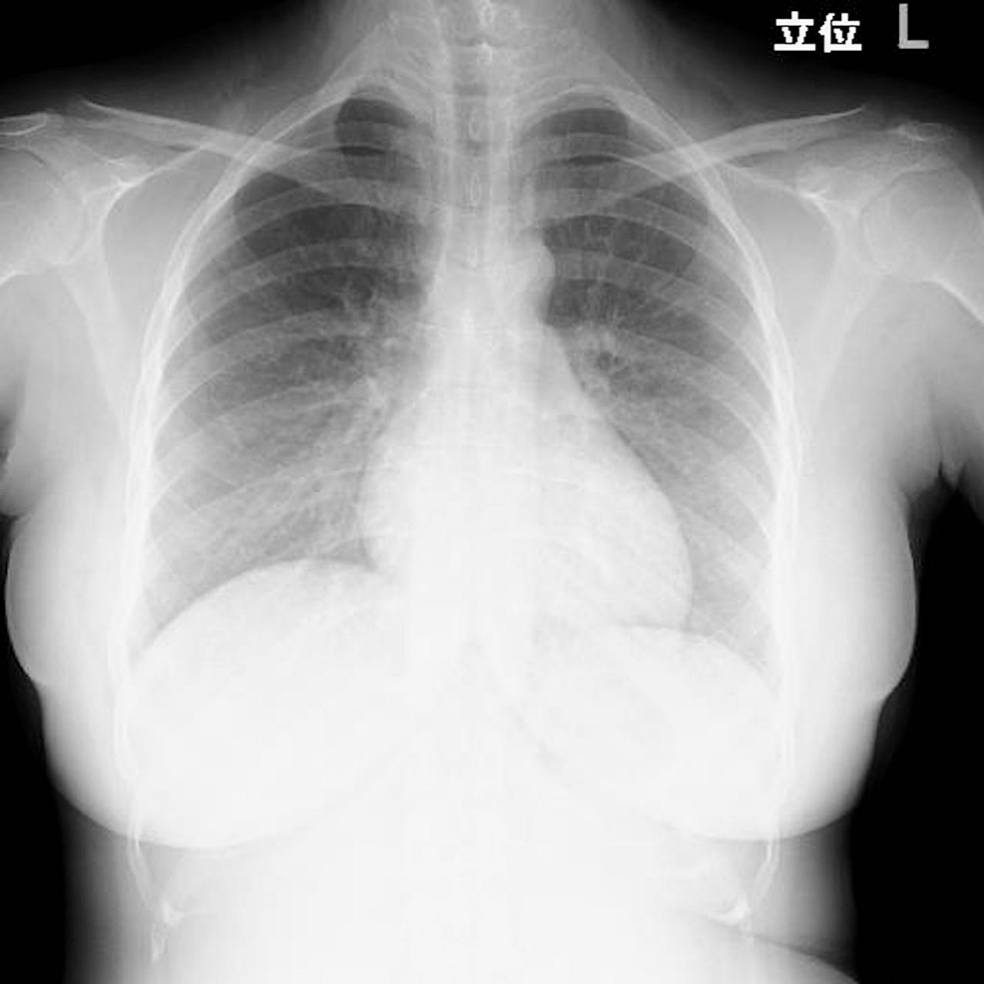
Chest X-ray image Normalized chest radiograph

Oxygenation gradually improved, and oxygen administration was completed on the fourth day of hospitalization. The patient was discharged home on the fifth day of hospitalization. Hb was 8.6 g/dl. Chest radiographs were normalized on discharge (Figure 3). Echocardiography performed three months after delivery showed that the LVD had shrunk to 50 mm, and MR was normalized.

## Discussion

In this case, EF was preserved on echocardiography and there was no evidence of diastolic dysfunction, but the diagnosis of heart failure was made based on decreased oxygenation, CT imaging findings of the lungs, and BNP exceeding 150 pg/ml [4].

HFpEF is diagnosed when the patient develops clinical symptoms of heart failure, and when echocardiography shows preserved EF but impaired diastolic function [5]. Peripartum cardiomyopathy is generally diagnosed by EF falling to less than 45% [2], but in this case, EF was preserved, so the possibility of HFpEF was considered. However, since the diastolic function was normal, HFpEF was not diagnosed. It has been reported that perinatal heart failure patients with preserved EF do not develop left ventricular enlargement [6]. Our case, in which left ventricular enlargement occurred with preserved EF and no diastolic dysfunction, is different from previous cases of perinatal heart failure.

The development of heart failure, in this case, may be related to the fact that the patient was anemic. Anemia has been reported as a prognostic factor for heart failure [7]. In addition, having a Hb level of less than 6 g/dl during pregnancy is associated with poor pregnancy outcomes [8]. In the present case, the patient's Hb level dropped to 5.3 g/dl due to heavy bleeding during delivery, and RBC 4U transfusion was performed, which improved the Hb level to 7.1 g/dl but did not normalize the situation. Therefore, anemia and perinatal heart failure may be related. However, we do not know whether anemia caused or exacerbated perinatal heart failure in this case. Therefore, future studies examining the association between anemia and postpartum heart failure are warranted.

## Conclusions

We experienced a case of postpartum heart failure with normal EF and diastolic function on echocardiography but with temporary left ventricular enlargement and MR. Since anemia is suspected to be associated with this case, future studies on anemia in cases of postpartum heart failure are warranted.
